# Flexible Molybdenum Electrodes towards Designing Affinity Based Protein Biosensors

**DOI:** 10.3390/bios6030036

**Published:** 2016-07-18

**Authors:** Vikramshankar Kamakoti, Anjan Panneer Selvam, Nandhinee Radha Shanmugam, Sriram Muthukumar, Shalini Prasad

**Affiliations:** 1 Department of Bioengineering, University of Texas at Dallas, 800 W. Campbell Rd., Richardson, TX 75080, USA; vxk121030@utdallas.edu (V.K.); axp107120@utdallas.edu (A.P.S.); nxr123230@utdallas.edu (N.R.S.); 2 EnLiSense LLC, 1813 Audubon Pond way, Allen, TX 75013, USA, sxm131031@utdallas.edu

**Keywords:** molybdenum, bioassay, flexible substrate, cardiac troponin-I, label-free biosensing

## Abstract

Molybdenum electrode based flexible biosensor on porous polyamide substrates has been fabricated and tested for its functionality as a protein affinity based biosensor. The biosensor performance was evaluated using a key cardiac biomarker; cardiac Troponin-I (cTnI). Molybdenum is a transition metal and demonstrates electrochemical behavior upon interaction with an electrolyte. We have leveraged this property of molybdenum for designing an affinity based biosensor using electrochemical impedance spectroscopy. We have evaluated the feasibility of detection of cTnI in phosphate-buffered saline (PBS) and human serum (HS) by measuring impedance changes over a frequency window from 100 mHz to 1 MHz. Increasing changes to the measured impedance was correlated to the increased dose of cTnI molecules binding to the cTnI antibody functionalized molybdenum surface. We achieved cTnI detection limit of 10 pg/mL in PBS and 1 ng/mL in HS medium. The use of flexible substrates for designing the biosensor demonstrates promise for integration with a large-scale batch manufacturing process.

## 1. Introduction

Point-of-care diagnostic devices offer efficient and cost-effective solutions for early detection of diseases and monitoring of patient health conditions [1]. Flexible polymers are the preferred choice of substrates in the point-of-care diagnostic biosensing devices owing to their enhanced physiochemical properties as well as integratability into multiple forms of consumer products. These flexible substrate-based biosensors hold promise for mass production thereby aiding in providing disease diagnostic capabilities to resource limited environments [2]. The signal response obtained is a result of affinity based binding between the surface immobilized recognition element and its target conjugate [3,4]. Affinity based biosensors consist of a biological recognition element such as an antibody or any other type of receptor immobilized on a sensor and integrated with a transducer to detect and measure the concentration of a target bio-analyte. Antibodies are the most widely used biological recognition elements in the affinity based biosensors due to their high affinity to proteins—also known as analytes—and commercial availability. The binding of the antibody to the antigen at the sensor surface generates a signal response. The phenomenon of detecting target analytes through the use of only one capture antibody that gives a distinguishable signal is called single-capture immunoassay [5].

The substrate of the sensor is a crucial component of the biosensor. Silicon has been used for the development of biosensors as it enables precise design of electrodes in the micrometric dimensions using microelectronic photolithographic processes and it favors the integration of signal processing hardware components on the same substrate [6]. The lack of flexibility of the silicon substrate is the key limiting factor for use in emerging applications such as flexible substrate biosensors. Flexible substrate biosensors can be scaled up to large-scale batch manufacturing processes and possess the advantages of low-cost and easy disposal [7,8]. A number of polymeric materials have been evaluated for the design of flexible biosensor platforms [9,10]. Porous nanomembranes are the preferred choice of substrate material in various biosensing applications [11,12]. One of the most favorable porous substrate polymers that has been used for flexible biosensors is polyamide [13,14,15]. The presence of pores in the substrate of the membrane based biosensors has been shown to enhance the signal response from the sensor due to the phenomenon of biomolecular nano-confinement [16,17]. The nanoporous membranes facilitate the elimination of charge screening caused by the non-specific components in the diffuse region of the electrical double layer by excluding the majority of macromolecules. The pores in the membrane are the sites where the biomolecular interactions occur, thereby resulting in an enhanced output signal. Enhanced signal response is obtained from the nanoporous electrodes with a pore size on the order of 200 nm when compared to planar electrodes for biosensing applications [18]. In addition, polyamide substrates offer commendable physical properties for liquid biosensing such as high mechanical strength and hydrophilicity.

Gold (Au) has been a preferred choice of electrode material in biosensing applications [19,20]. However, there is a growing interest in evaluating dichalcogenides for biosensing applications due to their high electron mobility and enhanced surface area to volume ratio [21,22]. Molybdenum (Mo) is transition metal with an electronegativity of 2.16 on the Pauling scale. It does not visibly react with oxygen or water at room temperature [23]. Many of the first-row transition elements have a known biological function, and in many cases redox reactions are linked to their role [24,25,26]. Molybdenum has been used as an bottom electrode material in bulk acoustic resonator applications due to its good electrical conductivity property [27]. Molybdenum electrodes have been demonstrated to form a thicker electrical double layer compared to nickel and platinum in the hydrogen production through water electrolysis in 1-butyl-3-methylimidazolium tetra fluoroborate (BMI.BF4) using electrochemical impedance spectroscopy (EIS) technique [28]. Electrochemical interfacial capacitance is defined as the capacitance per surface area and is a function of the electrical double layer capacitance [29]. Mo has been demonstrated to exhibit electrochemical behavior upon the interaction with an electrolyte [30]. The presence of interfacial capacitance of Mo can be leveraged for biosensing by enhancing the signal response from binding of biomolecules which is utilized in the EIS technique [28]. The binding between the biomolecule and the electrode surface is of crucial significance for the successful operation of the biosensor. Molybdenum demonstrates a favorable chemistry in binding with sulphur [31]. This property has been leveraged to form stable self-assembled monolayers between Mo and the cross-linker.

The biomolecule chosen for this study is a well-established cardiac biomarker, Troponin-I (cTnI), whose detection in the bloodstream signifies acute myocardial damage. Elevated levels of Troponin-I in the range of ng/mL or higher has been clinically correlated to the onset of myocardial infarction and other cardiac ailments. The enzyme-linked immunosorbent assay (ELISA) and radioimmunoassay (RIA) are conventional methods for monitoring the cTnI levels in a clinical environment [32,33]. The limit of detection associated with these conventional detection techniques are in the range of ng/mL to μg/mL. In order to detect an early spike in the levels of the cTnI, there is a need for biosensors for detecting lower concentrations of the cTnI in a reliable manner. Various biosensing techniques such as electrochemiluminescence [34], faradic electrochemical methods [35], and colorimetric methods [36] have been leveraged to detect the levels of the cTnI. Table 1 compares the performance matrices of various biosensing techniques.

Electrical biosensing is shown to be more robust than other label-free transduction mechanisms due to its speed, sensitivity, ease-of use, and low cost [40]. Electrical biosensing using EIS technique is a powerful method to monitor the events occurring on the electrode-electrolyte interfaces [41]. EIS technique has been evaluated and applied for the detection of number of bioanalytes [42]. In brief, EIS measurements can be performed in two ways: faradaic EIS and non-faradaic method. Faradaic impedance measurements are usually carried out by using a reversible redox probe, while non-faradaic impedance measurements are done without using any redox probe [43]. In a non-faradaic sensor, the capacitance of the electrode-electrolyte interface can be considered as the main indicator of interaction between the antibody and antigen [44]. The non-faradaic biosensors have the advantage of low instrumentation cost and have scope for miniaturization. Thus, a non-faradic biosensor based on EIS promises scope for development of a low-cost point-of-care diagnostic device for monitoring the levels of cTnI in a reliable manner.

Herein, we presented an easy and facile way to design an affinity based protein biosensor, which has immense scope for integration with a point-of-care diagnostic device. Conversely, to other previously reported works for cTnI detection, in this paper we demonstrate the detection of cTnI by employing the non-faradaic method to probe the cTnI concentration changes. We have utilized EIS technique to study the changes in the capacitance due to the interactions between molybdenum electrode and the protein biomarkers. We have demonstrated the feasibility of using molybdenum as an electrode material in biosensors for the detection of cardiac biomarkers. The electrochemical property exhibited by molybdenum upon its interaction with the electrolyte has been leveraged in the design of a non-faradic label-free electrochemical biosensor. The enhanced sensitivity obtained with the use of molybdenum as an electrode material is useful for accurate detection of concentration of cTnI. Thus, we have evaluated molybdenum as an electrode material in capturing signal response occurring at the sensor surface due to binding of biomolecules. The signal response is predominantly due to the charge perturbations in the electrical double layer that are transduced as changes in capacitance associated with binding of biomolecules.

## 2. Materials and Methods

### 2.1. Sensor Fabrication and Characterization

**Figure 1:**
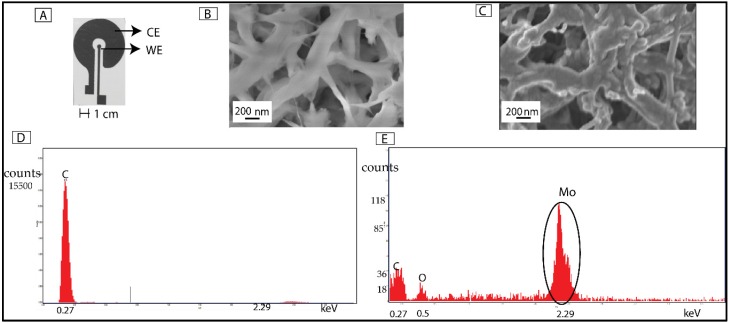
Material characterization of molybdenum (Mo) biosensor. (**A**) Representation of Mo sensor on polyamide substrate. CE represents the counter electrode and WE represents working electrode; (**B**) Representation of scanning electron microscope (SEM) image of Mo sensor on the porous polyamide substrate before Mo deposition; (**C**) Representation of SEM image of Mo sensor on the porous polyamide substrate before Mo deposition; (**D**) Representation of energy dispersive X-ray spectroscopy (EDAX) spectrum analysis on polyamide (PA) membrane before Mo deposition; (**E**) Representation of EDAX spectrum analysis on the Mo sensor. The encircled region on the EDAX spectrum indicates the peak corresponding to the L-shell energy peak of the Mo.

Molybdenum (Mo) electrochemical biosensors were fabricated using e-beam evaporation (99.9% purity Mo crucible) on nanoporous polyamide membrane substrates. Polyamide membranes (GE Healthcare Life Sciences, Pittsburgh, PA, USA) are flexible, lightweight and hydrophilic whose intercalated nanoporous structure allow the capillary wicking of test sample to the sensing region. The sensing region comprises two concentric circle electrodes which act as working and counter/reference electrodes. The design of the working and the counter electrodes is represented in Figure 1A. In a typical biosensing application, the biological molecule is immobilized on the working electrode and the signal resulting from the interaction of biological molecules is sensed from the working electrode. Thus, impedance of the counter electrode must be smaller. This is achieved by maintaining the area of the counter electrode at least ten times higher than that of working electrode [45]. In order to meet the above design requirement, the ratio between the counter and working electrodes’ area of the biosensor was designed to be 15:1.

The geometrical pattern of the designed electrodes was transferred on to substrate material using shadow masks with CHA Mark 50 e-gun evaporator in University of Texas at Dallas (UTD) cleanroom. The shadow masks were obtained from acrylic cellulose acetate sheets (Apollo^®^ Copier Transparency Film, Lincolnshire, IL, USA). The rate of deposition was maintained at 0.8 Å/s to achieve a uniform metal deposition and thickness of Mo deposition was maintained at 120 nm. The thickness of the deposition was validated through profilometric measurements. The conformal coating of Mo on polyamide were characterized using scanning electron microscope (SEM) and energy dispersive X-ray spectroscopy (EDAX). The morphological characteristics of material characterization is further discussed in the results section.

The measured resistivity of Mo deposition on polyamide with a 4-point probe source meter was 5.9 e^−4^ ohm cm while the resistivity prior to the Mo deposition was measured to be 6.6 e^−2^ ohm cm. The low resistivity of the molybdenum surface provides good electrical conductivity and thus is advantageous in achieving an enhanced sensor signal response for biosensing. The electrical contact to the potentiostat was established through alligator clips.

### 2.2. Surface Functionalization of Sensor

Dithiobis succinimidyl propionate (DSP) (Sigma-Aldrich, St. Louis, MO, USA) was dissolved in dimethyl sulfoxide (DMSO) (Sigma-Aldrich, St. Louis, MO, USA) to formulate a 10 mM mixture. Thirty microliters of the DSP-DMSO mixture was added to the Mo electrochemical sensor to allow functionalization of this thiol-based linker molecule on Mo surface and incubated for four hours. Phosphate-buffered saline (PBS) buffer (0.15 M) was added to the sensor to prepare the surface prior to addition of the antibody. Monoclonal anti-cTnI antibody stock solution was diluted to 1 μg/mL in PBS buffer and then immobilized on the DSP functionalized sensor surface and incubated for 15 min. The concentration of antibody to be used was determined through an antibody saturation study. The antibody saturation study experiment was conducted with varying antibody concentrations from 100 fg/mL to 1 μg/mL and the change in impedance with respect to a blank PBS sample was studied for the various antibody concentrations. The noise estimation after-antibody conjugation was studied by analyzing the impedance of the sensor for multiple PBS washes following the antibody conjugation. EIS measurements were taken after each assay step with Gamry Reference 3000 potentiostat (Gamry Instruments, Warminster, PA, USA) to validate the binding.

### 2.3. Calibration Dose Response Analysis for cTnI Detection

In order to evaluate the baseline sensor performance on an antibody conjugated sensor, blank buffer devoid of any antigen was added to the sensor. EIS measurements were performed after two minutes of addition of buffer. This measurement was considered as the zero-dose measurement. The impedance at the subsequent antigen concentrations was compared against the zero-dose impedance values. The cTnI antigen was diluted to the experimental concentrations in the target test buffer (i.e., PBS or human serum (HS)) (Fitzgerald, Acton, MA, USA). After the addition of antigen sample, the sensor was incubated for 15 min to allow for sufficient time for binding of antigen with the surface functionalized antibodies. EIS measurements were performed after the incubation time in order to validate the binding of antigen to the surface conjugated antibody. We tested the cTnI antigen concentration from 100 fg/mL to 10 μg/mL in PBS medium and from 100 pg/mL to 10 μg/mL in the HS medium. The dissociation constant between the cTnI antibody and the cTnI antigen was in the range of 10^−10^ M which corresponds to 2.4 pg/mL [46]. In order to validate the binding of the biomolecules on the porous substrate, we performed the negative control dose response experiment with Bovine Serum Albumin (BSA) (Sigma-Aldrich, St. Louis, MO, USA) protein.

### 2.4. EIS Technique for Label-Free Biosensing

**Figure 2:**
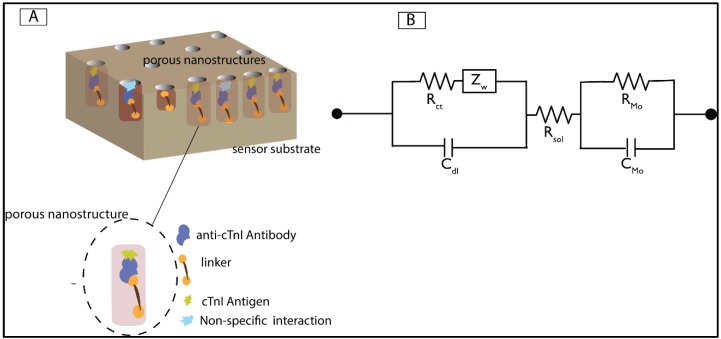
Biosensor design and electrical circuit model. (**A**) Schematic representation of immunoassay of the Mo biosensor indicating the deposition of Mo on the pores of the membrane. The enlarged segment indicates the building of immunoassay on the pores; (**B**) Schematic representation of the equivalent circuit of the Mo biosensor. C_dl_ represents the double layer capacitance dominated by biomolecular binding. R_sol_ represents the resistance contributed by the solution, Zw represents the Warburg Impedance and R_ct_ represents the charge transfer resistance, R**_Mo_** represents the resistance at the Mo electrode, C**_Mo_** represents the interfacial capacitance at the Mo electrode surface.

The technique of single capture immunoassay (primary antigen-antibody interaction in the absence of secondary antibody) was leveraged to achieve protein binding and subsequent detection process. The binding of the biomolecules onto the molybdenum electrode perturbs the inherent charge distribution in the electrical double layer (EDL). The perturbation in the charge distribution leads to capacitance changes in the EDL. Thus, the capacitance introduced by the bimolecular binding was measured by the EIS technique. The equivalent electrical circuit is depicted in the Figure 2B. The EIS technique used in this study is a modification of the standard electrochemical impedance spectroscopy technique wherein redox probes are used to study the interactions occurring at the surface probe. The absence of the use of the redox probe in the implemented sensing system makes it a non-faradic sensor. The electrical stimulus was applied across the electrode in order to direct the surface-charged biomolecules towards the sensing region of the biosensor. The resulting impedance is calculated using the voltage-time function equation as given below:

$$Z=\frac{V\left(t\right)}{I\left(t\right)}=\frac{V_{0}\sin\left(2\pi ft\right)}{I_{0}sin\left(2\pi ft+\phi \right)}$$

In the above equation, $V_{0}$ and $I_{0}$ represent the peak voltage and the current signals, ‘*f*’ represents the frequency of the applied signal, ‘*t*’ represents the time and ‘ϕ’ represents the phase shift between the voltage-time and the current-time functions. When the impedance measurement is carried over a spectrum of frequencies, the technique is referred to as impedance spectroscopy. The binding of the biomolecules causes a change in the output capacitance across the sensing region of the molybdenum biosensor. The output impedance consists of resistive and capacitive components. The capacitive component indicates the differential surface charge at the EDL as a function of antigen-antibody binding. The parameters of the input voltage—namely the amplitude of the sinusoidal voltage and the frequency of the input signal—need to be optimized in order to capture the changes in the impedance occurring as a result of binding of biomolecules. The alternating current (AC) input voltage is of the magnitude of 10 mV and the range of test frequency was varied from 100 mHz to 1 MHz. The application of input AC voltage causes the attraction of the ions in the solution towards the molybdenum electrode surface, which is known as inner Helmholtz plane (iHP). The outer Helmholtz plane (oHP) is constituted with ions which facilitate the functionalization of linker molecule onto the sensor surface. The length between the iHP and the oHP is regarded as the length of the EDL. The length of the electrical double layer extends from the iHP as the immunoassay is built on the sensor surface. Figure 2A represents the schematic representation for the immunoassay.

## 3. Results

### 3.1. Material Characterization of Mo Deposition

The material characterization of sensor surface performed with the SEM indicates the deposition of molybdenum on the porous polyamide substrate. Figure 1B,C shows the SEM images pre- and post-Mo deposition on polyamide membrane, respectively. The SEM micrograph post-Mo deposition validates the conformal deposition of Mo on the pores of the membrane substrate. The profilometry results post-Mo deposition validates the depth of the Mo deposited and its correlation with the preset value.

Figure 1D,E shows the EDAX spectra related to highlighted zone in the SEM micrographs for blank polyamide and Mo deposited polyamide. The objective of measuring the EDAX spectra was to investigate the presence of different elements on the substrate pre- and post-deposition. The significant peaks as observed in Figure 1D were for carbon and oxygen, which were due to the elemental composition of polyamide as a function of its hydrocarbon side chains. The EDAX spectra on blank polyamide substrate does not show any peaks correlating to the Mo. Post-deposition, the energy counts for carbon and oxygen were significantly reduced. The EDAX observed post-deposition showed highest energy counts for Mo. The distinct peak at an energy level 2.29 keV corresponds to the L-shell of Molybdenum thereby indicating its conformal deposition on porous polyamide. Polyamide favored rapid fluid wicking which facilitated the uniform distribution of sample solutions on the electrodes.

### 3.2. Baseline Electrical Characterization

The baseline sensor response refers to the study of material and electrical properties of the sensor in the absence of biomolecules. This study was performed in order to analyze the effect of inherent material and electrical properties of the Mo sensor. The baseline electrical properties of the Mo electrode biosensor was studied with EIS technique by the application of an AC voltage of 10 mV at 1000 Hz frequency as the electrode characteristics are studied as part of the bulk properties of the electrode which are studied at the high frequencies [42]. The lower frequencies reflect the effect of biomolecular binding on the electrical double layer. Hence the performance of the immunoassay was evaluated at 1 Hz frequency. The open-circuit impedance in the absence of any fluid on the sensor surface was measured to be 75 MΩ and the short-circuit impedance was measured to be 5224 Ω. A total of *n* = 3 replicates were performed and a CV of 8% was observed. The low percentage in the CV indicates that the baseline performance of the sensor stack was reliable for EIS biosensing.

### 3.3. Antibody Saturation Study

**Figure 3:**
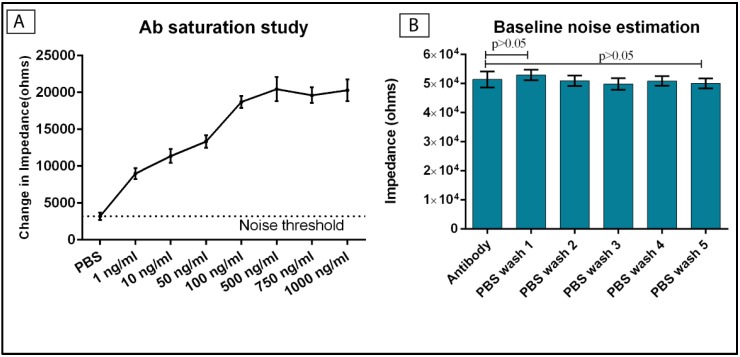
Antibody conjugation analysis. (**A**) Analysis of antibody saturation study on the dithiobis succinimidyl propionate (DSP) functionalized Mo sensor. The dotted line indicates the noise threshold. Error bars indicate the standard error of mean from *n* = 3 replicates; (**B**) Baseline noise estimation on antibody conjugated biosensor and multiple PBS wash steps post the Ab conjugation. The impedance values for the phosphate-buffered saline (PBS) wash steps are statistically insignificant compared to the impedance obtained post the antibody incubation.

The concentration of the antibody required to completely saturate the sensor surface functionalized linker sites is crucial in order to prevent competitive binding of free linker sites with other biomolecules. We performed the antibody saturation experiment in order to determine the concentration of antibody required for saturating the linker functionalized sensor surface. The experimental conditions for this study was set at 10 mV AC voltage and 1 Hz frequency. A total of *n* = 3 replicates of measurements were performed for this study and the results are represented in Figure 3A. The concentration of the antibody at which there is minimal change in impedance compared to the previous concentration is regarded as the saturating antibody concentration. The noise threshold was calculated from the difference in impedance value between the PBS step post-DSP functionalization and another blank PBS buffer dose step, and was determined to be approximately 3200 Ω. The change in impedance for the lowest antibody concentration of 1 ng/mL was measured to be 8900 Ω. The change in impedance for antibody concentration of 1000 ng/mL with respect to the baseline PBS measurement was approximately 20 kΩ. The change in impedance with respect to the previous concentration was negligible and hence 1000 ng/mL was the saturating concentration of the antibody required to completely saturate all the available linker sites for the antibody conjugation. Thus, the saturating dose concentration for the cTnI antibody was considered to be 1000 ng/mL. Figure 3B represents the results of the baseline sensor characterization for multiple PBS wash steps following the antibody conjugation. A total of *n* = 3 replicates were performed and a *p* value greater than 0.05 was obtained for all the PBS wash steps with respect to the impedance value obtained after antibody conjugation. Thus, the biosensor demonstrates stable measurements post-antibody conjugation after the buffer wash steps. The stable values in the impedance obtained after the PBS wash steps validates the conjugation of antibody to the linker sites and shows that the PBS wash steps does not dissociate the antibody from the sensor surface.

### 3.4. Calibration Dose Response Study

**Figure 4:**
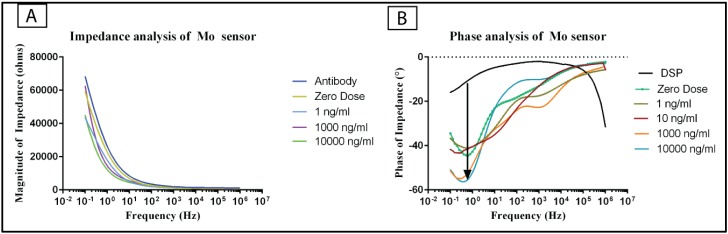
Magnitude and Phase analysis of Mo sensor. (**A**) Magnitude of impedance of Mo sensor. The magnitude of the impedance decreases with increasing antigen concentrations; (**B**) Phase analysis of Mo sensor. Maximum phase changes between the DSP functionalization step and subsequent assay steps were observed at 1 Hz frequency.

The calibration dose response study experiments were performed for a frequency range of 100 mHz to 1 MHz frequency with 10 mV (peak to peak) voltage. Figure 4A represents the change in the phase angle of the impedance as a function of frequency and Figure 4B represents the change in the modulus of impedance as a function of frequency. A lag in the phase between the input voltage and the output current is observed for the different assay steps. The phase lag is contributed by the change in the capacitive elements in the biosensor. The transient building of charges occurs at the electrical double layer as a result of biomolecular binding. The maximum phase for the DSP functionalized sensor was measured to be approximately 20°. The phase of the sensor increased with the subsequent assay steps due to increase in the capacitance at the molybdenum electrode due to the biomolecular binding. The phase of the system increased up to approximately 58° for the cTnI antigen with a concentration of 10,000 ng/mL. The changes in the phase with respect to the assay steps were significant. These significant changes in the phase indicate the presence of capacitive binding. The maximum changes in the phase between the DSP functionalization step and the subsequent assay steps were observed at 1 Hz frequency and hence the calibration dose response analysis were performed for impedance values at 1 Hz frequency. Since the maximum phase changes were observed at a frequency of 1 Hz, it is inferred that the capacitive behavior of the biosensor is dominant at this frequency. At high frequencies, the phase of the signal approaches zero for all the assay steps. Also, at higher frequencies, the impedance curves associated with the Bode magnitude analysis overlap with each other. Hence, the low frequency region (1 Hz) is chosen to study the distinguishability in the doses as a function of impedance. The impedance of the biosensor decreases with an increase in the frequency of the input signal. For instance, the impedance value at 100 mHz for 10 ng/mL is 45 kΩ and it decreases to 975 Ω at 1000 Hz frequency. The changes in the impedance for various concentrations of the antigen were distinguishable from each other. Thus, these distinct changes in the impedances for various antigen concentrations validate the binding between the surface functionalized antibody with the antigen molecule.

**Figure 5:**
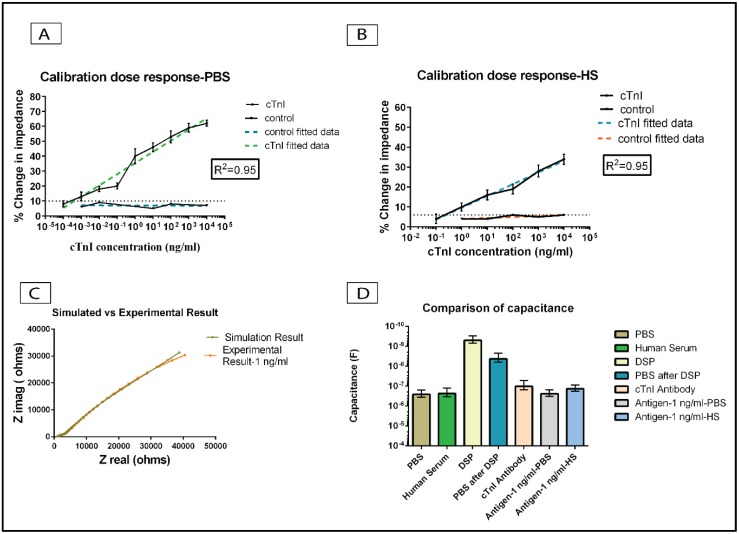
Calibration dose response analysis on Mo sensor. (**A**) Calibration dose response analysis in PBS medium. The dotted line indicates the noise threshold. The limit of detection (LoD) for the sensor in the PBS medium is 10 pg/mL; (**B**) Calibration dose response analysis in HS medium. The LoD for the sensor in HS medium is 1 ng/mL. Error bars represent the standard error of mean from *n* = 3 replicates; (**C**) Comparison of experimental vs. simulated result for 1 ng/mL of cTnI antigen; (**D**) Comparison of capacitance for various assay steps on Mo biosensor.

Figure 5A,B represents the results of calibration dose response experiments for cTnI protein detection in PBS and HS medium respectively at 1 Hz frequency. The percentage change in impedance with respect to the zero-dose for the lowest antigen dose of 100 fg/mL was 8%. The impedance difference with respect to the zero-dose measurement was calculated to be 3 kΩ. The percentage change in impedance increased with higher concentrations of the antigen. For the cTnI concentration of 10,000 ng/mL, the % change in impedance was approximately 60% and the change in impedance with respect to zero-dose sample was calculated to be 29 kΩ.

The control experiments with the BSA protein did not yield an increasing trend in the change in impedance percentage. The change in impedance with respect to zero-dose for the control doses were measured to be less than 10%. The change in the impedance for the control doses is attributed to the change in the diffusion driven impedance at the Mo electrode electrolyte interface.

The decrease in the impedance after the antigen binding is in correlation to the increase in the double layer capacitance due to the binding event. Thus, the percentage change in impedance increases with higher cTnI concentration. The increase in the change in the impedance for higher concentrations observed for the cTnI antigen concentrations indicate the signal from the sensor is a result of specificity in binding between the cTnI antigen and the cTnI antibody. The signal to noise ratio for an immunoassay assay is fixed at 3 [47,48]. Based on the analysis of the results of the calibration dose response, the difference in impedance between the impedance for dose concentration and that of negative control was measured to be 600 Ω, and this value is referred to as noise threshold for the calibration dose response study. The specific signal threshold (SST) is calculated as three times the noise impedance and was measured as 1800 Ω. In the Figure 5A, the percentage change in impedance for the cTnI concentration of 100 fg/mL was less than the noise threshold. The lowest concentration of cTnI antigen which was above the impedance at the noise threshold is regarded as the limit of detection (LoD) for the sensor. In our work, the LoD of the sensor is inferred to be at 10 pg/mL in PBS medium. The dynamic range of detection in the PBS medium was from 10 pg/mL to 10 μg/mL.

In order to simulate a diagnostic sensor environment, we tested the immunoassay in HS medium for cTnI detection. The experimental concentrations of the cTnI antigen were diluted in the human serum medium and tested on the antibody conjugated molybdenum electrode biosensor. The percentage change in impedance for the cTnI concentration of 100 pg/mL with respect to a zero-dose measurement with blank human-serum alone was measured to be 4%. This value lies within the noise threshold region. The lowest cTnI concentration which is above the noise threshold is 1 ng/mL. Hence the LoD for the sensor in the HS medium is 1 ng/mL. The change in impedance increased with an increase in the antigen concentration. The percentage change in impedance for 10,000 ng/mL cTnI concentration with respect to zero-dose measurement was measured to be 34%. The increase in the percentage change in impedance for increasing concentration of the antigen is an indication of increase in the double layer capacitance due to the binding of the biomolecules at the Mo electrode surface despite the presence of interfering biomolecules present in the human serum. The dynamic range of detection in the human serum medium was from 1 ng/mL to 10 μg/mL. The change in impedance for the control experiments with BSA protein were less than 7% with respect to zero-dose measurements. The negligible change in percentage for the negative doses indicates the specificity in the binding between the cTnI antigen and the cTnI antibody. The experimental data was fitted to a linear regression model for both control and cTnI antigen samples. R-square value of the calibration dose response curve was estimated as 0.95 for both PBS and human serum media. The experimental data results were compared with the simulated model derived from the electrical equivalent circuit parameters, and the comparison plots yielded a low chi-square value of 4.96 e^−6^. Figure 5C represents the comparison of Nyquist plot between the experimental result obtained for the 1 ng/mL cTnI antigen concentration and the simulated value. The simulated values were obtained after fitting the experimental data into the equivalent circuit as shown in Figure 2B. The simulated data points correlated to the experimental values with a low chi square value of 4.96 e^−6^. Thus, the presence of interfacial capacitance is validated in the derivation of equivalent electrical circuit for the biosensor. Figure 5D represents the comparison of experimentally measured capacitance values for various immunoassay conditions at Mo electrode. The measured capacitance increased from the DSP step to the antigen binding step thereby validating the capacitive binding between the biomolecules.

### 3.5. Optical Readout for cTnI Detection

**Figure 6:**
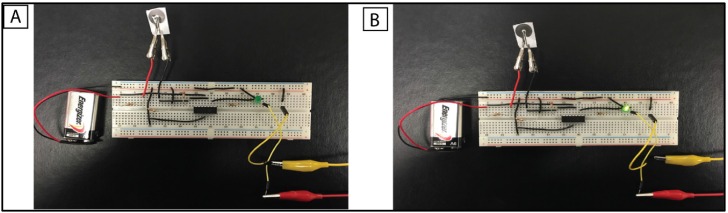
Electrical prototype of an optical reader for cTnI detection. (**A**) Optical reader indicating an “OFF” output state on an antibody conjugated sensor; (**B**) optical reader indicating an “ON” state upon the addition of antigen sample. The binding between of biomolecules decreases the impedance below the threshold value thereby turning the output LED to “ON” state.

In order to demonstrate that the sensing of cTnI molecules can be reported in a user-friendly format, we developed a LED based prototype to indicate the of cTnI antigen. Figure 6A indicates the cTnI antibody immobilized sensor. The LED prototype operates similar to a Boolean logic output indicating a change in the output for the impedance value correlating to the 10 pg/mL cTnI concentration. The simple LED prototype supports the favorability of molybdenum biosensor to be integrated with electrical circuit components. The threshold concentration to trigger the output response is preset as a correlation to an impedance value. The output LED glow is an indication of crossover point of the cTnI concentration. Figure 6B indicates ON state of the LED after the addition of cTnI antigen. The addition of the antigen on the antibody-immobilized sensor decreases the impedance of the circuit. A comparator integrated circuit compares the impedance with a preset threshold value and completes the circuit to make the LED glow. We observed that the output LED turned ON within 10 s of addition of the antigen of 10 pg/mL concentration. The rapid change in the output from the sensor indicates that Mo facilitates the charge conduction through the porous substrate. The reader can be extended to classify the cTnI concentration into multiple classification segments.

## 4. Discussion

We have leveraged the material properties of the molybdenum for use as an electrode material for the design of a cardiac biomarker (cTnI) detection biosensor. The flexibility of the polyamide substrate offers scope for integration of the biosensor with textile based point-of-care diagnostic platform. Polyamide membranes with a pore size of 200 nm were chosen for the application to primarily leverage the effect of size-based exclusion for reducing non-specific binding and enhancing signal response from specific biomolecular binding at the electrode-electrolyte interface. The presence of peaks in the EDAX spectrum analysis correlated with the energy levels of the Mo validates the conformal deposition on the polyamide substrate. SEM images of the Mo sensor represents the deposition of Mo on the porous polyamide membrane. The decrease in the impedance after the DSP incubation indicates that the Mo electrode surface exhibits affinity towards thiol linker molecule. The self-assembled monolayer formed at the sensor surface forms the stack upon which the biomolecules are immobilized. The linear increase in the change in the impedance of up to 20 kΩ for saturating antibody concentration of 1 μg/mL elucidates the binding between the antibody and the linker functionalized Mo electrode sensor surface. The increase in the change in the impedance is the results of the change in the double layer capacitance element at the interface of the Mo and the electrolyte. The phase of the biosensor increases up to 58° for the antigen concentrations. The increase in phase lag is due to capacitive binding at the pores of the polyamide surface.

The biosensor exhibited clear distinguishability in the impedances for various concentrations of the cTnI antigen at 1 Hz frequency. The change in the impedance is theoretically to the double layer capacitance element of the equivalent electrical double layer model (C_dl_). Molybdenum electrode facilitates the changes in the capacitance associated with the binding event between the surface conjugated antibody and the target antigen present in the sample. The changes in the impedance is predominantly driven by the changes in the capacitance occurring due to biomolecular binding. Mo electrode deposited on the porous polyamide substrate facilitates the capacitive binding between the biomolecules.

The biosensor showed a linear response over a broad detection range from pg/mL to μg/mL with approximately 60% change from the zero-dose impedance measurement in the PBS medium and a change of 35% in HS medium. The increase in the percentage change in impedance for higher sample concentrations validates the biomolecular binding. The lower limit of cTnI detection in the PBS medium is 10 pg/mL, as its change in impedance was higher than the noise threshold set by the control (BSA) protein concentrations. The lower limit of detection in the HS medium was 1 ng/mL. The small change in impedance for the negative control samples is attributed to the diffusion driven Warburg impedance (Z_w_). The increase in the measured capacitance with the immunoassay steps indicates that the molybdenum electrode drives the change in the double layer capacitance at the electrical double layer formed at the sensor surface thereby favoring the detection of precise changes in the cardiac troponin levels. The use of flexible substrate is advantageous for its scope for large-scale scalability in the biosensor fabrication. The sensors are disposable after one-time use and hence holds promise for integration with a point-of-care diagnostics device.

## 5. Conclusions

The electrochemical behavior of the Mo has been leveraged in building a label-free biosensor on a flexible membrane substrate. The biosensor demonstrates feasibility of cTnI detection in PBS and HS medium over clinically relevant concentrations for the cTnI detection. The efficient biomolecule detection capability of the molybdenum biosensor in the human serum, despite the presence of interfering biomolecules, supports the claim of molybdenum being an economically attractive alternative to gold as an electrode material in diagnostic biosensors. Thus, the molybdenum electrode biosensor has immense scope for use as a portable point-of-care diagnostic biosensor with the ability to detect the onset of cardiac disease in a reliable and rapid manner.

## Figures and Tables

**Table 1:** Comparison of immunoassay performance for cTnI detection.

Technique	LoD	Dynamic Range	Reference
Electrochemiluminescence	0.0025 ng/mL	0.0025–10 ng/mL	[34]
Faradaic Electrochemical Impedance Spectroscopy (EIS)	4.2 pg/mL	0.01–10 ng/mL	[35]
Optomagnetic biosensor	0.03 ng/mL	0.03–6.5 ng/mL	[37]
Colorimetric	0.01 ng/mL	0.01–5 ng/mL	[38]
Surface plasmon resonance	68 ng/L	68 ng/L–660 μg/L	[39]
